# P-117. Wastewater-Based Surveillance (WBS) of Shiga Toxin-Producing *Escherichia coli* (STEC) in Alberta, Canada

**DOI:** 10.1093/ofid/ofae631.324

**Published:** 2025-01-29

**Authors:** Barbara Jean M Waddell, Kristine Du, Emily Au, Jangwoo Lee, Nicole Acosta, Maria Bautista Chavarriaga, Rhonda Clark, John Conly, Linda Chui, Byron M Berenger, Thomas Griener, Jason Cabaj, Rebekah De Vinney, Xiaoli Pang, Bonita Lee, Christine O’Grady, Casey RJ Hubert, Michael Parkins

**Affiliations:** University of Calgary, Calgary, Alberta, Canada; University of Calgary, Calgary, Alberta, Canada; University of Calgary, Calgary, Alberta, Canada; University of Calgary, Calgary, Alberta, Canada; University of Calgary, Calgary, Alberta, Canada; University of Calgary, Calgary, Alberta, Canada; University of Calgary, Calgary, Alberta, Canada; University of Calgary, Calgary, Alberta, Canada; University of Alberta, Alberta Health Services, Edmonton, Alberta, Canada; University of Calgary, Calgary, Alberta, Canada; University of Calgary, Alberta Health Services, Calgary, Alberta, Canada; University of Calgary, Calgary, Alberta, Canada; Universtiy of Calgary, Calgary, Alberta, Canada; University of Alberta, Edmonton, Alberta, Canada; University of Alberta, Edmonton, Alberta, Canada; University of Calgary, Calgary, Alberta, Canada; University of Calgary, Calgary, Alberta, Canada; University of Calgary, Calgary, Alberta, Canada

## Abstract

**Background:**

Shigatoxin-producing *E. coli* (STEC) are responsible for significant human morbidity, with the potential to cause severe food-borne illness and outbreaks. STEC incidence varies between communities and peaks in summer months (PMID 31652648). Leveraging a SARS-CoV-2 WBS program, we sought to explore genomic targets for STEC WBS.

**Figure 1.**
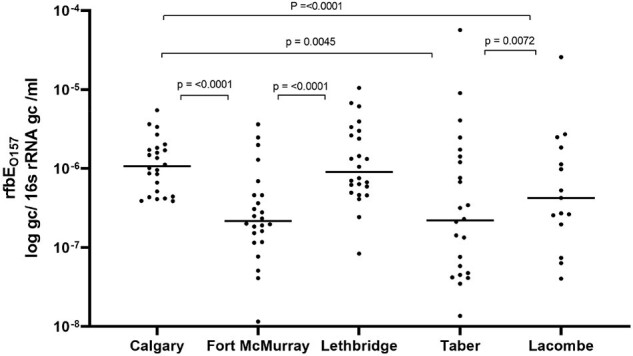
Prevalence of STEC differs across Alberta’s municipal sewer sheds

**Methods:**

Composite-24h wastewater (WW) was collected from geographically disparate, and socioeconomically diverse Alberta communities (n=5) at the level of municipal WW treatment plants. From 04/2022-03/2024, monthly WW underwent pelleting and DNA extraction by Qiagen DNeasy PowerSoil Pro kit. WW extracts were assessed for four potential genomic STEC targets: Shiga Toxin 1 (*stx1*), Shiga Toxin 2 (*stx2*), Intimin (*eae*) and LPS O antigen gene specific for O157 (*rfbE_O157_*), by multiplex PCR. Each target was normalized by 16S rRNA-for total bacterial burden. WW STEC targets were assessed for correlation using Spearman’s and compared between communities and seasonality (July-Sept vs Jan-Mar) by Mann-Whitney U-test.

**Figure 2.**
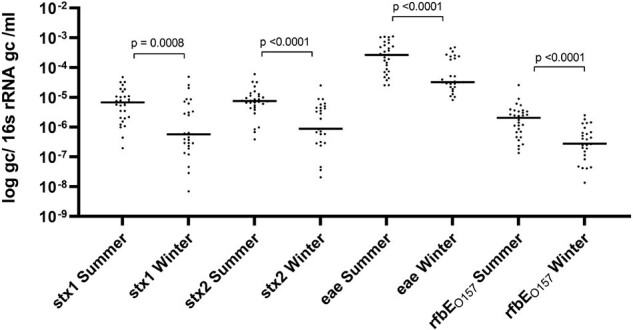
STEC WBS demonstrates seasonal prevalence trends

**Results:**

Of 111 WW samples assessed, 108 (97%) were positive for all targets, and individual targets were identified in 110 (99%) *stx1;* 110 (99%;) *stx2;* 111 (100%) *eae*, and 110 (99%) *rfbE_O157_*. Gene abundance for each STEC target exhibited strong correlations across sites (*stx1* vs *stx2*, r=0.802, p< 0.0001; *stx2* vs *rfbE_O157_*, r=0.634, p< 0.0001; *eae* vs *rfbE_O157_*, r =0.551, p< 0.0001; *stx2* vs. *eae* r=0.542, p< 0.0001). WW measured STEC gene targets exhibited significant differences between municipalities (**Figure 1**) with strong seasonal trends (**Figure 2**).

**Conclusion:**

WBS for STEC yielded patterns consistent with established patterns of disease (PMID 31652648). All four STEC genomic targets demonstrated significant correlation across sewersheds. STEC WBS may represent a novel tool to understand and monitor population-level activity and prevent disease.

**Disclosures:**

**All Authors**: No reported disclosures

